# Physically Active Men Show Better Semen Parameters than Their Sedentary Counterparts

**DOI:** 10.22074/ijfs.2017.4881

**Published:** 2017-09-03

**Authors:** Paula C. Lalinde-Acevedo, B. Jose Manuel Mayorga-Torres, Ashok Agarwal, Stefan S. du Plessis, Gulfam Ahmad, Ángela P. Cadavid, Walter D. Cardona Maya

**Affiliations:** 1 Reproduction Group, Department of Microbiology and Parasitology, Medical School, University of Antioquia, Antio- quia, Colombia; 2 Center for Reproductive Medicine, Cleveland Clinic, Cleveland, Ohio, USA; 3 Division of Medical Physiology, Faculty of Medicine and Health Sciences, Stellenbosch University, Tygerberg, South Africa; 4 Department of Physiology and Cell Biology, University of Health Sciences, Lahore, Pakistan

**Keywords:** Sperm, Fertility, Physical Activity, Sedentary, Lifestyle

## Abstract

**Background:**

The quality of semen depends upon several factors such as environment, life style, physical activity, age, and occupation. The aim of this study was to analyze and compare the conventional and functional semen parameters in men practicing vigorous physical activity to those of sedentary men.

**Materials and Methods:**

In this descriptive cross-sectional study, semen samples of 17 physically active men and 15 sedentary men were collected for analysis. Semen analysis was performed according to the World Health Organization (WHO) guidelines, while functional parameters were evaluated by flow cytometry.

**Results:**

Results showed that several semen parameters (semen volume, viability, progressive motility, total motility, normal morphology, and moribund cells) were superior in the physically active group in comparison with the sedentary group. Semen parameters such as viability, progressive motility and total motility, as well as the percentage of moribund spermatozoa were significantly different between both groups. However, sperm DNA damage, lipid peroxidation and mitochondrial potential were not significantly different among the groups.

**Conclusion:**

Nevertheless, the physical activity shows better semen parameters than sedentary group. Taken together, our results demonstrate that regular physical activity has beneficial impact in sperm fertility parameters and such a life style can enhance the fertility status of men.

## Introduction

The conventional semen analysis involves the macroscopic (volume, pH, and colour) and microscopic (motility, concentration, viability, and morphology) examination (1). It reflects the secretory activity of the testes, epididymis and accessory sex glands indirectly (2). Although conventional semen analysis provides both quantitative and qualitative information, it does not include evaluation of the functional properties of spermatozoa (3-7). Furthermore, oxidative stress which may directly contribute to the origin of male infertility, is not measured (8). Oxidative stress occurs due to the imbalance between the reactive oxygen species (ROS), reactive nitrogen species (RNS), and seminal antioxidant reserve in the male reproductive tract (9, 10). These ROS or RNS are produced during normal cellular metabolism and can be from either endogenous (normally produced by oxidative phosphorylation in mitochondria) or exogenous origin (e.g. produced by leukocytes) (10, 11).

Physiological levels of ROS exert a critical role in spermatozoa, triggering and mediating important signaling events to acquire essential functions such as hyperactivation, capacitation, and acrosome reaction (10-12). However, an excess in ROS levels is detrimental to cellular function and spermatozoa are highly susceptible to oxidative stress due to a lack in repair mechanisms (8, 13). This may result in damage to the structural components of the axoneme which may impact on the motility patterns (14, 15). It may also induce lipid peroxidation of cellular membranes (16), thereby disrupting the fluidity of mitochondrial and plasma membranes (12, 17) and furthermore lead to oxidative damage to proteins involved in the fusing of the spermatozoon with the oolemma (15). Additionally, ROS may cause DNA damage due to impaired histone remodeling during sperm maturation (12). Oxidative damage to spermatozoa has been related with recurrent pregnancy loss (13, 18, 19) and male infertility (5, 20).

It is well known that certain environmental factors including prolonged and continued exposure of the whole body, testes or scrotum to: i. Increased temperature, even at 37°C (21-23), ii. Environmental pollutants and endocrine disruptors (21, 24), iii. Electromagnetic radiation (21, 25), as well as lifestyle factors such as smoking, recreational drug use, alcohol consumption, obesity and sedentary occupation or lifestyle (25-29), may influence sperm quality and male fertility potential (21, 23, 25) mediated by induction of oxidative stress leading to cell apoptosis. Among several life style factors, sedentarism have been found associated with several medical conditions and considered as one of the main causes of major public health issues at present (30). According to the definition of Bernstein et al. (31), individuals are considered sedentary when they spend less than 10% of their daily energy expenditure on performing moderate to vigorous-intensity activities. Also a sedentary person frequently spends much time sitting or lying down and performing activities usually associated with this low energy consumption state such as sleeping or watching television. Also it is commonly avoiding any form of exercise or sporting activities (32). Over the past five decades, changes in the occupational activities and leisure time have promoted the sedentary behavior and impacted on lifestyle (33, 34). As is true for other medical conditions (obesity and heart diseases), this phenomenon is equally deleterious for semen quality (35).

On the other hand; physical activity has beneficial effect on human health and is defined as any voluntary and repetitive body movements produced by skeletal muscle action that substantially increases energy expenditure above the basal state (34, 36). It may be included in the occupational activities or have diverse purposes like being aerobic training or training strength, flexibility and balance, therefore it encompasses exercise and sport (37, 38). Physical activity is classified according to the intensity with which it is practiced and may be quantified in terms of the energy expenditure as a multiple of the resting metabolic rate (39). Using the Metabolic Equivalent of the Task (MET, a physiological measure expressing the energy cost of physical activities), the moderate physical activity producing noticeable accelerated heart rate, ranges from 3.0 to 6.0 MET while the vigorous physical activity, demanding greater physical effort causing rapid breathing and a substantial increase in heart rate, are all the physical activities above 6.0 MET (38, 39). Some studies have reported a positive relationship (27, 33, 40-44), while others reported a negative one (45- 47) between the practice of physical activity and the semen quality. Others report no impact of physical activity on sperm quality (48-51), therefore, the effect of a physically active lifestyle to improve semen quality is still controversial (52). The present study was conducted with the specific aim to evaluate and compare the semen parameters, conventional as well as functional, of men practicing vigorous physical activity to those having a sedentary life style.

## Materials and Methods

In this descriptive cross-sectional study, thirty two men of reproductive age (physically active group 27.5 ± 6.0 and sedentary group 26.6 ± 5.3 Lalinde-Acevedo et al. years) from Medellin, Colombia were included. The inclusion criteria were: healthy men, without testicular disease, with a body mass index (BMI)<26 kg/m^2^, and those who followed the same lifestyle pattern for the 12 months preceding the study, it is, either be physically active group (PAG, practice vigorous physical activities with a >6 MET for more than 2 hours per occasion at least 3 times per week; activities included are cycling, stationary cycling, calisthenetics, weightlifting, dancing, running, marital arts, football and swimming) or be sedentary group (SG, minimal physical activity ≤3 MET, do not practice sportive activities) (39, 53) (Table 1). Recreational drug and anabolic steroid users, smokers or medicated men were excluded from the study. Ethical approval was obtained from the Research Ethics Committee of the University of Antioquia and all patients gave informed consent. Semen samples were collected from volunteers between January and July 2014 by masturbation after a recommended ejaculation abstinence of 3-6 days. In addition to sample collection, certain anthropometric measurements (height and weight) necessary to calculate BMI were also measured (Table 1).

Also Participants had to complete a self-administered questionnaire by providing information regarding their reproductive history and whether or not they routinely practice any physical activity. If they did, they were asked to fill the description, type, frequency, intensity and duration of the physical activities practiced. This information was used to calculate the physical activities MET using the “Compendium of physical activities” as proposed by Ainswort et al. (39) which provided a measurement of their intensity level.

### Conventional semen analysis

After complete liquefaction of the semen samples (30-60 minutes, at 37°C), a basic semen analysis was performed according to the World Health Organization (WHO) guidelines (1) while the sperm concentration was determined by using a Makler chamber (Seﬁ-Medical Instruments, Haifa, Israel) (54). Finally, sperm morphology was analyzed following the Tygerberg strict criteria (55), and semen samples with leukocytospermia (>1×10^6^ white blood cells/mL) were excluded.

### Functional analysis

All flow cytometry analysis reported in this study were conducted on an Epics XL flow cytometer (Becton Dickinson, CA, USA) with a 488 nm excitation wavelength supplied by an argon laser. Forward scatter and side scatter measurements were used to gate spermatozoa’ and exclude debris’ and aggregates limiting undesired effects in the overall fluorescence. All data were acquired and analyzed using WinMDI 2.9 Software (Scripps Research Institute, La Jolla, CA) and a total of 10000 events were collected per sample.

### Mitochondrial membrane potential

Mitochondrial membrane potential (ΔΨ_m_) was measured by using 3, 3′-dihexyloxacarbocyanine iodide stain (DIOC_6_, Molecular Probes Inc., The Netherlands) (3) a cationic lipophilic dye selective for the mitochondria of living cells. Propidium iodide (PI, Molecular Probes Inc., The Netherlands) was used as counter stain to discriminate necrotic/dead cells. Briefly, 2×10^6^ spermatozoa were incubated in 300 μL of phosphate buffer saline (PBS, pH=7.4) containing DIOC_6_ (final concentration of 10 nM) and PI (final concentration of 12 μM) in the dark (30 minutes, at 25°C). Then, samples were washed in PBS (180 x g, 5 minutes), the pellet re-suspended in PBS and subjected to flow cytometry. Data were acquired as the percentage of living spermatozoa showing high (ΔΨ_m high_) or low (ΔΨ_m low_) green fluorescence and dead spermatozoa-red fluorescence.

### Intracellular reactive oxygen species production

The intracellular ROS and RNS (specifically H_2_O_2_, HO^-^, ROO^-^ and ONOO^-^) levels were evaluated using 2’, 7’-dichlorodihydrofluorescein diacetate (DCFH-DA, Sigma-Aldrich, St Louis, MO, USA). Upon cleavage of the acetate groups by intracellular esterases, DCFH is selectively oxidized by the above mentioned ROS and RNS to the green fluorescent DCF. PI was used to exclude the necrotic/ dead cells (5). DCFH-DA was diluted to a final concentration of 1 μM in 300 μL of PBS containing 2×10^6^ spermatozoa and PI (final concentration 12 μM). The cell suspensions were incubated in the dark for 5 minutes, at 25°C, washed three times with PBS (180 x g, 5 minutes) and the pellet re-suspended in PBS before being analyzed by flow cytometry. Results are expressed as the percentage of live spermatozoa exhibiting the green DCF fluorescent response (DCF positive spermatozoa), as well as the green media fluorescence intensity (MFI).

### Plasma membrane integrity evaluation

The LIVE/DEAD® Sperm Viability Kit (Molecular Probes Inc., The Netherlands) which distinguishes three populations of sperm based on their staining patterns, was used to assess the integrity of the plasma membrane according to the manufacturer’s instructions. Briefly, 2×10^6^ spermatozoa were incubated in 300 μL of PBS with Sybr-14 and PI (green and red fluorescence emission, final concentration of 1 μM and 12 μM, respectively) in the dark (30 minutes, 25°C), washed once and re-suspended in PBS prior to flow cytometry analysis. Data are expressed as the percentage of viable spermatozoaintact plasma membrane cells (positive to SYBR-14 and negative to PI), necrotic/dead cells (positive for PI only) or moribund sperm (positive for both dyes).

### Lipid peroxidation assay

Oxidative degradation of lipids was measured using the BODIPY (581/591) C11 (Molecular Probes Inc., The Netherlands) according to the method proposed by Aitken et al. (16). BODIPY (581/591) C11 once incorporated into sperm membranes, undergoes a fluorescent emission shift from orange to green upon peroxidation by ROS. Briefly, 2×10^6^ spermatozoa suspended in 300 μL of PBS were incubated in the dark (30 minutes, at 25°C) with BODIPY C11 (final concentration 6.6 μM), washed and re-suspended in PBS before flow cytometry analysis. Results are expressed as the percentage of spermatozoa exhibiting the green fluorescence response.

### Sperm Chromatin Structure Assay

The Sperm Chromatin Structure Assay (SCSA) was used to determine the sperm DNA fragmentation index by Evenson (56) as previously described and modified in our laboratory (5, 13, 18, 19). Briefly, 400 μl of acid detergent solution (HCl, NaCl, Tritón X-100, water, pH=1.2) were added to 10×10^6^ spermatozoa suspended in 200 μL of TNE buffer (TRIS-HCL, NaCl and EDTA, pH=7.4). After 30 seconds, spermatozoa were stained with 600 μl of acridine orange (Sigma-Aldrich, St Louis, MO, USA) staining solution (final concentration of 6 μg/mL). The ratio of single stranded DNA (red) to single plus double stranded DNA (green) MFI was expressed as the DNA fragmentation index (DFI).

### Statistical analysis

The distribution of the data was evaluated with the normality test of residuals. The t test was used to compare groups of data that assumed Gaussian distribution, while the Mann-Whitney test used to compare the variables that did not assume Gaussian distribution. Correlations between sperm variables were determined with the Pearson correlation coefficient. Data were analyzed by using Prism 5.0 (GraphPad Software, San Diego, CA) statistical software and a P<0.05 considered to be significant. Data following Gaussian distribution are expressed as the mean ± SD and those not assuming Gaussian distribution are expressed as median and range.

## Results

According to the MET scores, men were stratified into a physically active group (PAG, 8-48 MET, n=17) and a sedentary group (SG, <3 MET, n=15). Both PAG and SG present similar characteristics with regards to abstinence (4.1 ± 0.69 vs. 3.7 ± 0.75 days), height (1.74 ± 0.06 vs. 1.72 ± 0.05 m) and BMI (23.7 ± 1.5 vs. 22.7 ± 1.8 kg/m^2^). The average weight was slightly higher in the PAG in comparison with SG (71.6 ± 7.3 vs. 67 ± 5.3 kg), because these men had increased body mass in the form of muscle not of fat (Table 1).

**Table 1:** Characteristics of the participants


Characteristic	Physically active group n=17	Sedentary group n=15	P value

Age (Y)	27.5 ± 6.0	26.6 ± 5.3	0.66^+^
Sexual abstinence (days)	4.1 ± 0.69	3.7 ± 0.75	0.56^+^
Weight (Kg)	71.6 ± 7.3	67 ± 5.3	0.07^+^
Height (m)	1.74 ± 0.06	1.72 ± 0.05	0.3^+^
BMI (Kg/m^2^)	23.7 ± 1.5	22.7 ± 1.8	0.11^+^
Metabolic Equivalent of the Task (MET)	19.7 ± 10.6	1.8 ± 0.6	<0.0001^^^


Results are expressed as mean ± SD. BMI; Body mass index, +; Student t test (Gaussian distribution), and ^; Mann-Whitney test (non-gaussian distribution).

All semen samples from the PAG appeared normal with regards to viscosity and showed no agglutination, however various samples from SG showed moderate to high viscosity (33%) as well as isolated agglutination (47%) and moderate to abundant agglutination (13%) respectively. Among the conventional sperm parameters, total sperm motility, progressive motility and the percentage of viable sperm were significantly higher (P<0.05) in the PAG compared to the SG (Table 2). The only functional parameter that showed significant difference (P<0.05) between the PAG and SG was the percentage of moribund spermatozoa (Table 3).

**Table 2:** Conventional semen parameters


Variable	Physically active group n=17	Sedentary group n=15	P value

Semen volume (mL)	4.3 ± 1.2	3.5 ± 1.5	0.14^+^
Sperm concentration (×10^6^ sperm/mL)	95.2 ± 47	114.4 ± 63.9	0.37^+^
Total sperm count (×10^6^)	353.6 (55.72-1080)	361.9 (100-997.4)	0.82^^^
Viability (%)	80.2 ± 7.2	71.9 ± 10.7	0.01^+^
Progressive motility (%)	63.0 (55.7-87.7)	56.8 (35.2-82.7)	0.03^^ ^
Non-progressive motility (%)	3.7 (1.6-22.0)	5.0 (2.7-16.6)	0.13^^^
Total motility (%)	66.5 (70.0-89.3)	62.3 (42.5-45.6)	0.03^^^
Normal morphology (%)	7.3 (2.3-12.0)	4.8 (2.7-13.4)	0.52^^^
Abnormal head (%)	90.2 ± 5.0	89.1 ± 4.7	0.54^+^
Abnormal neck/middle piece (%)	44.9 ± 16.0	53.9 ± 18	0.14^+^
Abnormal tail (%)	5.1 (3.3-7.1)	6.9 (2.5-8.7)	0.52^^^
Abnormal cytoplasmic droplets (%)	6.6 ± 4.8	5.7 ± 2.9	0.56^+^


Values are expressed as mean ± SD in data with normal distribution, and median (range) in non-normal distribution. +; Student t test (Gaussian distribution) and ^: Mann- Whitney test (non-gaussian distribution).

**Table 3:** Functional seminal parameters


Variable	Physically active group n=17	Sedentary group n=15	P value

∆Ψm high spermatozoa (%)	63.5 (51.5-80.6)	63.8 (18.9-77.1)	0.45^^^
∆Ψm low spermatozoa (%)	4.0 (1.9-14.0)	4.4 (2.3-19.1)	0.54^^^
Sperm with intact plasma membrane (%)	68.1 (43.7-83.1)	65.2 (26.2-72.6)	0.10^^^
Moribund sperm (%)	4.3 (2.0-17.0)	9.0 (4.6-14.4)	0.02^^^
Necrotic/dead sperm (%)	23.5 ± 6.7	28.6 ± 11.4	0.13^+^
DCF positive spermatozoa (%)	59.3 (6.83-78.2)	49.3 (14.3-68.1)	0.09^^^
DCF positive spermatozoa (MFI)	50.6 (24.4-148.8)	57.7 (14.6-92.2)	0.74^^^
Sperm with lipid peroxidation (%)	3.3 (0.5-18.8)	6.3 (0.15-33.6)	0.20^^^
DNA fragmentation index (%)	19.6 ± 8.6	17.1 ± 8.3	0.40^+^


Values are expressed as mean ± SD in data with normal distribution and median (range) in non-normal distribution. DCF; 2', 7'-dichlorofluorescein, MFI; Mean fluorescence intensity, +; Student t test (Gaussian distribution), and ^; Mann-Whitney test (non-gaussian distribution).

When comparing the combined data sets from both groups, significant correlations were found between total abnormal sperm forms and spermatozoa with head defects (correlation coefficientr-= 0.67, P<0.01), sperm with neck/middle piece defects and progressive motility (r=-0.56, P<0.01), ejaculation abstinence time and sperm with excess residual cytoplasm (r=0.58, P<0.01), ejaculation abstinence time and non-progressive motility (r=-0.57, P<0.01), viable sperm and intracellular ROS production (r=0.79, P<0.01), viable sperm and sperm with high mitochondrial membrane potential (ΔΨ_m_, r=0.83, P<0.01), and sperm with high ΔΨ_m_ and intracellular ROS production (r=0.65, P<0.01). In addition, when the PAG’s data were analyzed separately, all of the above mentioned significant correlations were found, together with a few significant correlations exclusive to the PAG. These include: ejaculation abstinence time and immotile sperm (r=-0.57, P<0.01), sperm concentration and normal morphology (r=0.72, P<0.01), and sperm concentration and sperm with head defects (r=-0.63, P<0.01).

## Discussion

found differences in conventional and functional seminal parameters between physically active group and sedentary group of men. The semen parameters were better in PAG, which is in favor to adopt such a life style. The average values of the conventional parameters analyzed for each group, remained above the lower limit reference values proposed by the WHO (1). The total and progressive sperm motility, sperm viability, as well as the percentage of moribund cells were significantly higher in the PAG compared to SG. This is the first study in addition to conventional semen parameters, certain sperm functional parameters i.e. ΔΨ_m_, plasma membrane integrity, intracellular ROS and lipid peroxidation, were analyzed in relation to the practice of vigorous physical activity or following a sedentary lifestyle. Our results are in accordance with previous study demonstrating increased sperm motility in physical active men (41) and comparable results in a group of assisted reproduction patients classified according to their physical status (43). However, significant differences in sperm viability due to physical activity levels had not been previously reported.

Some studies reported that sperm concentration and morphology are the main parameters improved in men having moderate to vigorous physical active lifestyle (47), against being sedentary (43). Our results are not in agreement with these findings, as we did not observe any significant differences in either sperm concentration or morphology between PAG and SG. Nonetheless, a positive correlation was found between sperm concentration and normal morphology in PAG. Similar results have been reported by Munuce et al. (57) in semen samples obtained from men attending a reproductive clinic without regarding their physical status. This finding is interesting because it may be related to an increase in hormones, specially folliclestimulating hormone (FSH), luteinizing hormone (LH) and testosterone, responsible to stimulate proper spermatogonia nutrition and division during the process of spermatogenesis (58, 59). This speculation is supported by the findings from previous studies where increased total and free blood plasma and serum testosterone, as well as higher FSH and LH levels have been demonstrated after continuous moderate physical activity (41).

The plasma membrane integrity is a key determinant for proper sperm interactions with other cells and their environment, therefore it is a prerequisite for successful fertilization (60). The percentage of dual stained (moribund) spermatozoa was statistically higher in SG in comparison with PAG. This sperm population have been described as slightly damaged sperm with compromised plasma membrane that have lost their ability to exclude PI, indicating a transitional phase in which the cell ultimately die (60-62). Although the biological importance of moribund sperm has not been well established, in works on bulls the percentage of moribund spermatozoa was positively correlated with the low fertility status of males, possibly compromising the availability of live sperm in the female reproductive tract (62). Furthermore, Garner and Johnson (61) have microscopically observed that the change from green to red fluorescence of some sperm, began at the posterior portion of the sperm head, proceed anteriorly and is accompanied by the progressive loss of motility until they are dead. This increased percentage of moribund spermatozoa and negative correlation between sperm with neck/middle piece defects and progressive motility in SG can be the possible explanations of significantly lower progressive and total motility in these men.

The evaluation of functional parameters has also been used to determine the levels of oxidative stress in spermatozoa. Common sedentary activities such as sitting for long, and some physical activities including running or bicycling may disrupt the intrascrotal temperature regulation (63, 64) and increase the pressure force to the testicles (46, 47, 49), leading to oxidative stress (58).

Although the percentage of DCF positive spermatozoa in the PAG group was higher in SG, it was not significantly different. We found higher values of DCF positive sperm in comparison with previous studies (65-67) intended to evaluate the ROS/RNS production on spermatozoa using the same method. However; the oxidative stress level as depicted by lipid peroxidation measurement was discernibly lower in PAG than SG, which is in accordance with previous findings by others (16, 68). Sperm DNA integrity did not differ significantly in our study between PAG, SG and DFI remained in the range considered normal (16-24%) (69). This is in accordance with a previous report where no relation of sperm DFI was drawn in men with sedentary lifestyle in relation to their BMI and their waist circumference (70).

In addition, no mitochondrial dysfunction was detected either in the PAG or SG group despite the total and progressive motility is significantly increased in PAG. In fact, most of the spermatozoa in the semen samples from both groups had high ΔΨ_m_, which is indicative in proper mitochondrial functioning (17, 71). It may support the assumption that the rapid transition from viable to moribund sperm was influencing the loss of motility in the SG sperm rather than the viable sperm that have diminished the ΔΨ_m_ as it may be commonly related. As the higher ROS detection in the PAG was not correlated with oxidative stress generation in spermatozoa (higher lipoperoxidation-LPOand/ or altered DFI), we speculate that there must have been a balance between pro-oxidants and antioxidants molecules in the PAG volunteers’ semen samples. Possibly the practice of vigorous physical activity of volunteers, have contributed to attenuate the oxidative stress events in consequence of the higher ROS/RNS production, since it has been previously demonstrated that physical training promotes blood total antioxidant capacity (72), and also in semen, moderate to vigorous physical activity practitioners had superior levels of antioxidant enzymes in comparison with high performance-elite athletes or sedentary men (73).

Furthermore; it is known that sperm cells have a deficient ROS-scavenging system, in consequence of its limited cytosolic space. So they are very dependent on the antioxidant protection provided by the male reproductive tract (74). This is directly influenced by the men’s nutritional status and the dietary intake of antioxidant molecules since they form an essential part of the human antioxidant defense system (75).

As we did not control the diet in our volunteers, the effect of the diet cannot be ruled out, considering that, a physically active lifestyle is commonly accompanied by a healthy diet. In the light of these results, we consider convenient to include some other informational aspects, certainly related to the physically active or sedentary lifestyle and the semen quality. For instance, nutritional aspects related with dietary antioxidants intake, the determination of blood hormonal levels (mainly LH, FSH and testosterone) and the semen total antioxidant capacity evaluation, directly involved in the developmental environment of spermatozoa and the oxidative stress dynamics. On the other hand, it has also been established that if the physical training is at least moderate but regular, it may turn into an adaptation to diminish the increased amounts of ROS producing during high oxygen consumption derived from further vigorous physical activities (41, 58, 76, 77). This physical activity linked-adaptation constitutes an advantage over the possible adverse conditions associated with the practice of some previously mentioned physical activities that may negatively affect the seminal quality.

Infertility affects approximately 15% of couples of reproductive age, with significant impact on their quality of life (11, 70). As it is estimated that men contribute equally (50%) to the causes of fertility problems (29, 59), the identification and modification of some potential risk factors such as the relationship between physical activity or inactivity and semen quality, may help some couples to achieve their reproductive goals (70). The practice of vigorous physical activity is clearly not the unique solution. Most of the literature regarding the relatioship between physical activity, sedentarism and semen quality, have focused on elite athletes or men attending fertility clinics. However; various investigations have demonstrated the positive influence of moderate, constant exercise on the hormonal profile (41, 58, 76), libido (78), the psychological wellbeing (59) and on the body condition (30, 38, 76), which may also impact positively the male reproductive outcome. Our volunteers may be clasified as recreational but vigorous physical activity practitioners, since none of them were endurance sport competitors and the activities performed included strenght and aerobic training or vigorous occupational physical activities. This is important to clarify because the type of physical training, specially the higher intensity or constantly anaerobic training have been related to diminishseminal parameters (45-47, 49) and also may influence the hormonal effect on the sperm quality, specially on the testosterone metabolism (41, 59).

## Conclusion

Despite the fact that some indicators of cellular oxidative stress were higher in the PAG in comparison with the SG, no signs of developing a state of oxidative stress was observed. On the contrary, the practice of vigorous physical activity in the conditions set in our study (8 to 48 MET, in sessions of two hours minimum, with a frequency of at least 3 days a week), was significantly related to better semen parameters (increased viability, progressive and total motility and lower percentage of moribund cells), when compared to individuals following a complete sedentary lifestyle at least for a year. It can therefore be concluded that the levels of physical activity reported in this study, exert a positive effect on the semen parameters of these men or at least prevent its deterioration as a result of environmental stressors. Our findings are encouraging since they contribute to elucidate the proper intensity and frequency of physical activity which may excerpt a positive effect on semen quality or at least prevent its decline related to the practice of higher intensity-endurance physical activities. Future studies are required in defining the intensity and threshold to be considered as beneficial for semen quality.
